# Improved Charge Separation in WO_3_/CuWO_4_ Composite Photoanodes for Photoelectrochemical Water Oxidation

**DOI:** 10.3390/ma9050348

**Published:** 2016-05-07

**Authors:** Danping Wang, Prince Saurabh Bassi, Huan Qi, Xin Zhao, Gurudayal, Lydia Helena Wong, Rong Xu, Thirumany Sritharan, Zhong Chen

**Affiliations:** 1 Singapore-Berkeley Research Initiative for Sustainable Energy (SinBeRISE), CREATE Tower, 1 Create Way, #11-00, 138602 Singapore; dpwang@ntu.edu.sg (D.W.); RXu@ntu.edu.sg (R.X.); ASSRITHARAN@ntu.edu.sg (T.S.); 2 School of Materials Science and Engineering, Nanyang Technological University, 50 Nanyang Avenue, 639798 Singapore; PRINCE1@e.ntu.edu.sg (P.S.B.); QIHU0002@e.ntu.edu.sg (H.Q.); xinzhao@ntu.edu.sg (X.Z.); GURU0007@e.ntu.edu.sg (G.); 3 School of Chemical and Biomedical Engineering, Nanyang Technological University, 62 Nanyang Drive, 637459 Singapore

**Keywords:** CuWO_4_, WO_3_, composite thin film, charge separation, photoelectrochemical water splitting

## Abstract

Porous tungsten oxide/copper tungstate (WO_3_/CuWO_4_) composite thin films were fabricated via a facile *in situ* conversion method, with a polymer templating strategy. Copper nitrate (Cu(NO_3_)_2_) solution with the copolymer surfactant Pluronic^®^F-127 (Sigma-Aldrich, St. Louis, MO, USA, generic name, poloxamer 407) was loaded onto WO_3_ substrates by programmed dip coating, followed by heat treatment in air at 550 °C. The Cu^2+^ reacted with the WO_3_ substrate to form the CuWO_4_ compound. The composite WO_3_/CuWO_4_ thin films demonstrated improved photoelectrochemical (PEC) performance over WO_3_ and CuWO_4_ single phase photoanodes. The factors of light absorption and charge separation efficiency of the composite and two single phase films were investigated to understand the reasons for the PEC enhancement of WO_3_/CuWO_4_ composite thin films. The photocurrent was generated from water splitting as confirmed by hydrogen and oxygen gas evolution, and Faradic efficiency was calculated based on the amount of H_2_ produced. This work provides a low-cost and controllable method to prepare WO_3_-metal tungstate composite thin films, and also helps to deepen the understanding of charge transfer in WO_3_/CuWO_4_ heterojunction.

## 1. Introduction

In recent years, photoelectrochemical (PEC) water splitting has aroused tremendous research interest due to its sustainable and carbon neutral attributes for producing high energy density fuels [1,2,3,4]. A visible light active photoanode which utilizes more of the solar spectrum is highly desirable, and thus the earth-abundant α-Fe_2_O_3_ (band gap 2.1 eV) and WO_3_ (band gap 2.6 eV) have become popular candidates for water oxidation. Due to the intrinsic long-range antiferromagnetic order in α-Fe_2_O_3_, it has a short hole diffusion length (2–20 nm) [5,6] and low charge-carrier mobility (10^−2^–10^−1^ cm^2^·V^−1^·S^−1^) [7]. In comparison, WO_3_ has a much longer hole diffusion length (150 nm) and higher mobility (10 cm^2^·V^−1^·S^−1^) [8,9]. Nevertheless, its limited utilization of the visible light spectrum and its chemical instability in neutral electrolyte call for further improvement of WO_3_ for use as photoanode material [10,11,12].

Copper tungstate (CuWO_4_) has recently been recognized as a promising tungsten-based ternary oxide for PEC water oxidation [9,13,14,15,16,17,18,19,20]. It is an n-type semiconductor with a smaller band gap of 2.3 eV, corresponding to a theoretical photocurrent density (J_max_) of 10.7 mA/cm^2^ [18,21,22]. In addition, CuWO_4_ is stable in neutral pH electrolyte, because of the hybridization of Cu-d orbitals with O-p orbitals leading to higher chemical stability due to strong metal-oxo bonds [9,16,23,24]. Recently, several studies reported on the preparation of single phase of CuWO_4_ photoelectrodes and evaluation of their PEC properties for water oxidation. For example, CuWO_4_ thin films prepared by electro-deposition shows a photocurrent density of 0.20 mA/cm^2^ in a linear sweep test at the applied bias of 1.23 V *vs.* reversible hydrogen electrode potential (V_RHE_) in pH 7 potassium phosphate buffer solution [9]. CuWO_4_ formed by addition of excessive amounts of Cu(NO_3_)_2_ solution onto porous WO_3_ thin film followed by annealing exhibited approximately 0.15 mA/cm^2^ at 1.23 V_RHE_, and good stability in pH 7 (phosphate) and pH 9 (borate buffer) solutions [20]. CuWO_4_ thin films prepared by co-sputtering Cu and W metals on FTO glass displayed a photocurrent density of 0.20 mA/cm^2^ at 1.23 V_RHE_ [25].

However, the empty orbital of Cu(3dx^2^-y^2^) in pure CuWO_4_ is detrimental to charge transfer in the pure phase [16]. Therefore, fabrication of CuWO_4_-based composites or heterojunction photoanodes is needed to improve its charge transfer properties. For example, CuWO_4_:WO_3_ composite electrode fabricated by simultaneous electrochemical deposition with CuWO_4_:WO_3_ at a 1:1 ratio can oxidize water at a faster rate than pure CuWO_4_ electrode of similar thickness [15]. Another CuWO_4_/BiVO_4_ heterojunction photoanode prepared by spray-coating a Bi:V (1:1) precursor solution onto electrodeposited CuWO_4_ thin film led to a nearly 1.8-fold increase of the photocurrent density compared to pure CuWO_4_ [16]. Solid solution electrodes of CuWO_4_:CuMoO_4_ (CuW_1-x_Mo_x_O_4_) were synthesized by simultaneous electrochemical deposition of CuWO_4_ and CuMoO_4_ with different W:Mo ratios. These solid solution electrodes exhibited enhanced photocurrent and the incident photon-to current efficiency (IPCE) compared with pure CuWO_4_, which is possibly due to the lowered conduction band edge caused by the presence of molybdenum atoms, thus leading to a reduced band gap [26]. In addition, noble metals such as gold and silver nanoparticles when mixed in CuWO_4_ film increase the speed of charge transfer in the thin films [27,28].

As Cu^2+^ can react with WO_3_ to form CuWO_4_ during high temperature annealing [20], it is possible to fabricate WO_3_/CuWO_4_ composite or heterojunction thin films with controlled addition of Cu^2+^ precursor onto WO_3_ substrate. There are a few recent studies on the preparation of WO_3_/CuWO_4_ composite thin films [29,30,31], but quantitative analysis of light absorption and charge separation efficiency and definite experimental evidence of PEC gas evolution remain elusive.

In this paper, we prepared WO_3_/CuWO_4_ heterojunction thin film via a facile *in situ* conversion method. Controllable loading of CuWO_4_ could be achieved with different runs of dip coating. Moreover, with the addition of organic surfactant F127, the formed CuWO_4_ layer contained interconnected porous nanostructures. These WO_3_/CuWO_4_ films showed enhanced PEC performance over individual WO_3_ or CuWO_4_. By decoupling the photo absorption and charge transfer processes, it is shown that WO_3_/CuWO_4_ heterojunction thin films exhibit enhanced efficiency in each process because of the narrower band gap of CuWO_4_, and favorable band alignment between WO_3_ and CuWO_4_. Electrochemical impedance spectroscopy indicated a reduced charge transfer resistance for the composite thin film under AM1.5 illumination with 1.2 V_RHE_ bias. Further, H_2_ and O_2_ evolution test was undertaken, and the photocurrent generated from water splitting was confirmed by the detection and quantification of the product gases. This *in situ* conversion strategy can be used to fabricate other porous WO_3_/metal tungstate composite thin films. Our results also provide some reference for designing photoanodes with higher efficiency.

## 2. Results and Discussion

### 2.1. Synthesis and Characterization of Pristine WO_3_ and WO_3_/CuWO_4_ Composite Thin Films

**Figure 1:**
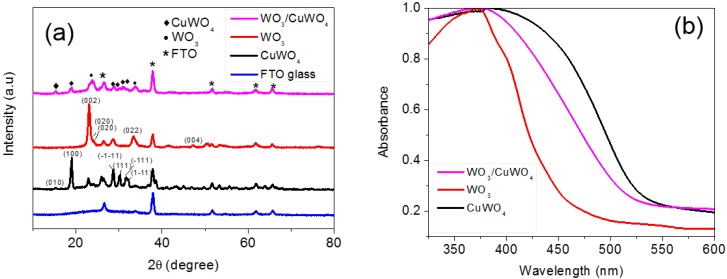
XRD patterns (**a**) and UV-Vis absorption spectrum (**b**) of pristine WO_3_, WO_3_/CuWO_4_, and CuWO_4_ thin films.

In this report, three representative samples with 0, 4 and 8 runs of programmed dip coating of Cu^2+^ solution are discussed, which gave rise to WO_3_, WO_3_/CuWO_4_ composite and CuWO_4_ materials respectively. XRD patterns in Figure 1 indicate that the WO_3_ thin films obtained from magnetron sputtered metallic W thin film are of the monoclinic phase (JCPDS No. 43-1035), which is the most photocatalytically active phase for water oxidation [10]. For comparison, we also sputtered metallic W onto a normal glass slide, which produced the orthorhombic phase of WO_3_ hydrate (JCPDS No. 35-0270) [10], to the contrary, as shown in XRD patterns in Figure S1. It is thus believed that the crystalline layer of F:SnO_2_ helped with the formation of monoclinic phase of WO_3_. Thin films of WO_3_/CuWO_4_ and CuWO_4_ showed the characteristic peaks of CuWO_4_ (JCPDF 72-0616) [9]. UV-Vis/DRS results of the three samples are shown in Figure 1b. Pristine WO_3_ absorbs light up to 464 nm, which is in accordance with the band gap of about 2.7 eV of monoclinic WO_3_. The light absorption of CuWO_4_ extends to 540 nm, corresponding to the smaller band gap of CuWO_4_ (2.3 eV).

**Figure 2:**
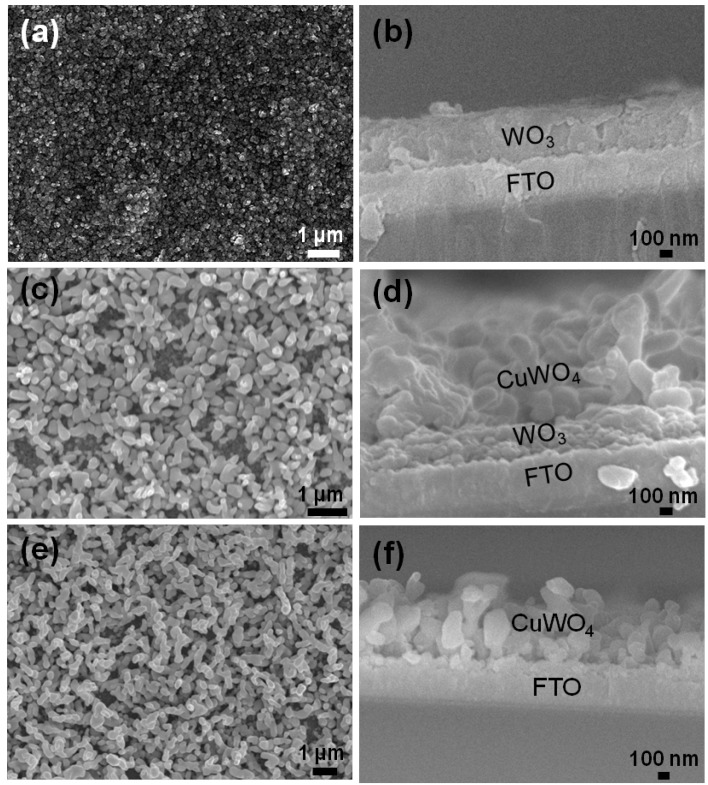
FESEM images of top and cross-section views of WO_3_ (**a**,**b**); WO_3_/CuWO_4_ composite (**c**,**d**) and CuWO_4_ (**e**,**f**) thin films.

The top and cross-section views of all the films observed by field emission scanning electron microscope (FESEM) are shown in Figure 2. A summary of the average thickness of WO_3_ and CuWO_4_ of each sample is provided in Table 1. Figure 2a,b shows the images of pristine WO_3_. The WO_3_ thin film was composed of densely assembled WO_3_ nanoparticles of irregular shape around 50–90 nm in diameter, and the average thickness of film was around 415 nm. Figure 2c,d are the images of WO_3_/CuWO_4_ composite thin film, with a porous layer of CuWO_4_ particles uniformly grown on the WO_3_ substrate. The thickness of WO_3_ layer was greatly reduced to 150 nm due to the *in situ* reaction with Cu^2+^ ions to form CuWO_4_. The transmission electron microscope (TEM) image of the scraped particles from the composite film in Figure S2 displays a network-like structure in the thin film, corresponding to the porous CuWO_4_ nanostructure in FESEM observation. In Figure 2e,f, with further increased loading of Cu^2+^ ions, the WO_3_ film was completely consumed, forming a porous layer of CuWO_4_ 1 μm thick.

### 2.2. Photoelectrochemical Performance of Thin Films

**Figure 3:**
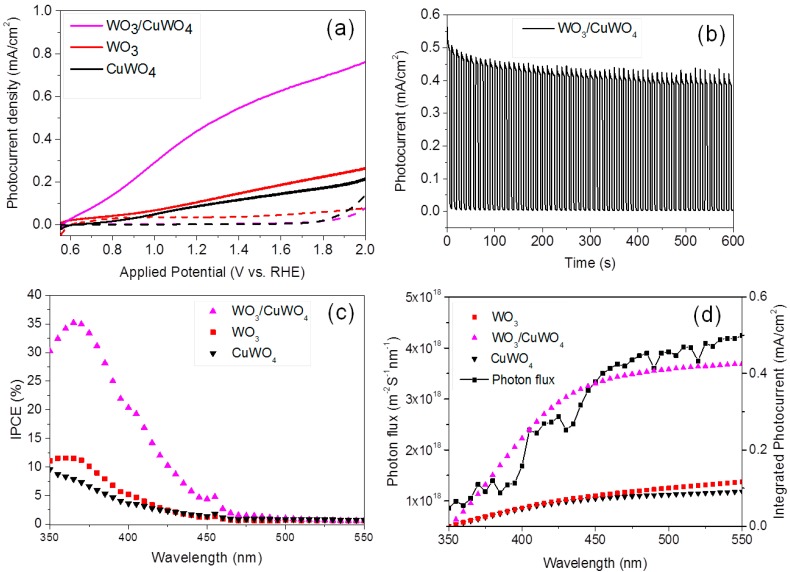
(**a**) Linear sweep voltammetry of WO_3_, WO_3_/CuWO_4_ and CuWO_4_ electrodes (solid lines: photocurrent under AM 1.5G illumination, dashed lines: dark current); (**b**) Photocurrent stability of WO_3_/CuWO_4_ electrode at 600 s; (**c**) IPCE measurement of WO_3_, WO_3_/CuWO_4_, and CuWO_4_ electrodes (note: IPCE was done using back illumination which is in accordance with photocurrent-potential measurement); (**d**) Integrated photocurrent based on the IPCE data (350–550 nm), solar photon flux is shown as a reference. (**a**–**c**) were tested in 0.5 M of Na_2_SO_4_ aqueous solution with illumination of AM 1.5; and (**b**,**c**) had 1.20 V_RHE_ bias applied to the electrodes.

The pristine WO_3_, WO_3_/CuWO_4_, and CuWO_4_ thin films were used as photoanodes in a conventional three-electrode setup. Their PEC performance was investigated by measuring the photocurrent with back-illumination as shown in Figure 3. In the linear sweep voltammetry (LSV) results in Figure 3a, all the samples exhibited negligible photocurrent under dark condition. Composite thin films obtained from different runs are provided in Figure S3. At 1.20 V_RHE_, bare WO_3_ and CuWO_4_ were of very similar photocurrent density, and WO_3_/CuWO_4_ (0.45 mA/cm^2^) showed a current density more than two times higher than WO_3_ and CuWO_4_ electrodes. As shown in the supporting information (Figure S3), all the composite thin films demonstrate higher photocurrent compared to single component thin films. The relatively low photocurrent of the WO_3_ underlayer in our report could be due to the preparation condition and insufficient thickness. As we annealed the tungsten film in air to let it oxidize into WO_3_, the insertion of oxygen atoms will make the thin film expand. As a result, the back contact with fluorine doped tin oxide (FTO) could be adversely affected. In addition, the annealing temperature and time can also affect the oxygen vacancies and carrier density, which will subsequently influence the photocurrent of WO_3_ films. However, when a thin layer of WO_3_ was coupled with CuWO_4_, the photocurrent of the composite film was remarkably improved compared with the single phase. Figure 3b shows the on-off photocurrent profile of the composite WO_3_/CuWO_4_ under a constant bias of 1.20 V_RHE_, recorded over a duration of 600 s with interval of 5 s. The composite thin film showed a constant photocurrent though transient spikes were spotted for both samples, which could be caused by the surface-trapped photo-generated minority carriers which recombine with the photo-generated major carriers [32]. Figure 3c shows the IPCE curves for WO_3_, WO_3_/CuWO_4_ and CuWO_4_ electrodes measured at 1.20 V_RHE_. The WO_3_/CuWO_4_ composite thin films had much higher IPCE values compared with WO_3_ and CuWO_4_ films. Though light absorption of CuWO_4_ covered the wavelengths up to 540 nm, the identical wavelength ranges of the three thin films in the IPCE curves indicate that the charge carriers generated in CuWO_4_ in the wavelength range 470–540 nm make little contribution to photocurrent generation [29,31]. The simulated photocurrents in Figure 3d for all samples were also calculated by integrating the IPCE spectra with a standard AM 1.5 G solar spectrum from Equation (1). The simulated photocurrent is independent of the light source and applied filters, and thus it is more accurate in evaluation of the PEC performance of the thin films. The values obtained were reasonable compared to measured photocurrents.

(1) $$I_{\mathrm{simulated}}=\int_{350}^{550}\left(IPCE\left(\lambda \right)\times \Phi \left(\lambda \right)\times e\right)d\lambda$$

where λ is the wavelength of light in unit of nm and IPCE (λ) is measured and calculated as will be described in Experiment Section 3.5. Φ(λ) is the photon flux of sunlight in photons/m^2^/s. The photon flux can be measured from tabulated solar irradiance data, E(λ), via Φ(λ) = E(λ)/(1240/λ) [33].

**Figure 4:**
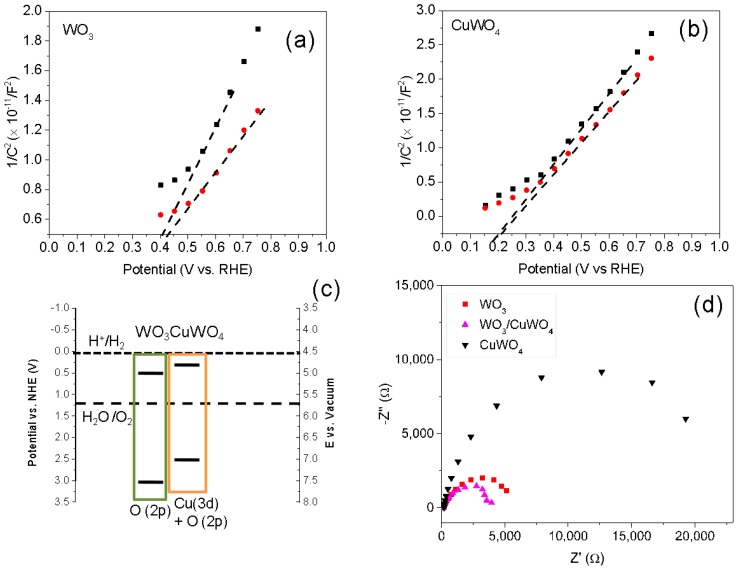
Mott-Schottky plots of WO_3_ thin film (**a**); and CuWO_4_ thin film (**b**) at 10 k and 5 k Hz under dark conditions; (**c**) Band structure of WO_3_ and CuWO_4_; (**d**) Nyquist plots of WO_3_, WO_3_/CuWO_4_, and CuWO_4_ thin films under AM 1.5 illumination in 0.5 M Na_2_SO_4_ with 1.20 V_RHE_ applied bias.

In order to measure the band structures of WO_3_ and CuWO_4_, the Mott-Schottky measurement is shown in Figure 4a,b, and the result for WO_3_/CuWO_4_ composite thin film is shown in Figure S4. Given the band gap measured from UV-Vis absorption, the band structures of WO_3_ and CuWO_4_ are shown in Figure 4c. The conduction band of CuWO_4_ is located at +0.2 eV (*vs.* normal hydrogen electrode, NHE) and that of WO_3_ is located at +0.4 eV (*vs.* NHE), while valence band of CuWO_4_ is at +2.4 eV (*vs.* NHE), lower than that of WO_3_ at +3.0 eV (*vs.* NHE). Therefore, these two components can form a heterojunction pair, and photo-generated holes from the inner WO_3_ layer will be transferred to the outer layer of CuWO_4_ in WO_3_/CuWO_4_ composite electrode.

The carrier density can be calculated by

(2) $$\frac{1}{{C_{\mathrm{SC}}}^{2}}=\frac{2}{e\varepsilon \varepsilon_{0}N_{D}}(E-E_{\mathrm{fb}}-\frac{K_{b}T}{e})$$

where C_SC_, q, ε_0_, ε, N_D_, and E_fb_ are capacitance, the electron charge, permittivity in vacuum, dielectric constant, donor carrier density and flat-band potential of the semiconductor, respectively. Using Equation (2), the carrier densities are 1.73 × 10^19^ for WO_3_ and 3.36 × 10^18^ for CuWO_4_. Our low carrier density is possibly due to the synthesis method for the thin films. The literature has shown that annealing in air can affect both the oxygen vacancies and the types of other vacancies/defects within a nanostructured thin film [28].

Electrochemical impedance spectroscopy (EIS) measurements were carried out to evaluate the overall resistance of the three photoanodes, and is shown in Figure 4d under illumination and 1.2 V_RHE_ bias. The semicircle in the medium-frequency region was attributed to the charge-transfer process. The diameter of the WO_3_/CuWO_4_ semicircle was the smallest among the three electrodes, which was in accordance with the LSV results [16,25]. CuWO_4_ had the largest semicircle diameter, indicating a very high charge transfer resistance in the thin film, which was possibly related to the poor intrinsic charge transfer property of CuWO_4_. However, when CuWO_4_ was coupled with WO_3_ to form a heterojunction composite anode, the photogenerated electrons in CuWO_4_ could be transferred to the WO_3_ underlayer, with good charge transport characteristics, and contribute to the reduced resistance. Thus, the composite electrode combined the excellent charge transfer characteristics of WO_3_ and good light absorption capability of CuWO_4_.

### 2.3. Comparison of Absorption Efficiency (η_abs_), and Charge Separation Efficiency (η_sep_) of WO_3_, CuWO_4_ and WO_3_/CuWO_4_ Thin Films

Since the WO_3_/CuWO_4_ electrode demonstrated remarkable improvement in photoelectrochemical and EIS measurement compared with pristine WO_3_ substrate and CuWO_4_ films, we further extracted the efficiency values of light absorption (η_abs_) and charge separation (η_sep_) based on Equations (3) and (4) to quantify the contribution of each factor [34,35].

(3) $$J_{abs}=\int_{350}^{550}(\eta_{abs}\;\times \Phi \left(\lambda \right)\times e)d\lambda$$

(4) $$\eta_{sep}=J_{sca}/J_{abs}$$

where J_abs_ is the photo current density calculated by multiplying η_abs_ by the standard AM 1.5 G (100 mW/cm^2^) solar spectrum, Φ(λ) is the photon flux of sunlight in photons/m^2^/s, e is the charge of an electron (C). J_sca_ is the photocurrent of the photoelectrode in the presence of scavengers as a function of applied bias.

**Figure 5:**
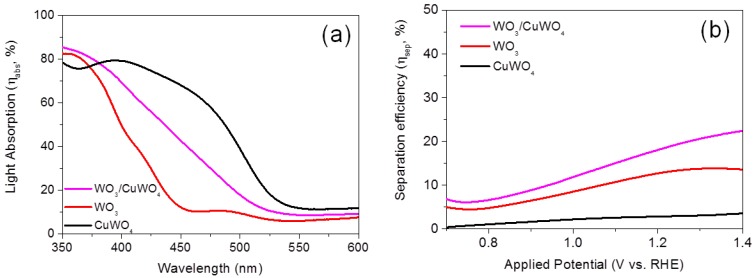
(**a**) Light absorption efficiency of WO_3_, CuWO_4_ and WO_3_/CuWO_4_ films obtained from an integrating sphere; (**b**) Charge separation efficiency of WO_3_, CuWO_4_ and WO_3_/CuWO_4_ electrodes.

By measuring the light transmittance and reflectance in an integrated sphere (see Figure S5), we were able to obtain the η_abs_ of pristine WO_3_, CuWO_4_ and WO_3_/CuWO_4_ in Figure 5a. It shows that more photons can be absorbed in the presence of CuWO_4_. With the measured values of η_abs_ and Equation (1), the integrated J_abs_ over the AM 1.5 spectrum of pristine WO_3_, CuWO_4_ and WO_3_/CuWO_4_ films are 1.8, 4.7 and 3.2 mA/cm^2^, respectively. The charge separation efficiency (η_sep_) can be determined by adding the hole scavenger H_2_O_2_ to the electrolyte. The presence of H_2_O_2_ increased photocurrent density of all the three thin films (Figure S6), which was due to the much faster charge transfer rate promoted by the hole scavenger. According to Equation (4), charge separation (η_sep_) efficiency of the three films was obtained and shown in Figure 5b. The composite WO_3_/CuWO_4_ thin film showed significant improvement of charge separation efficiency compared with CuWO_4_ film. This indicates that with a thin underlayer of WO_3_, the charge separation characteristics of CuWO_4_ are greatly enhanced, which leads to much higher photocurrent.

### 2.4. Photoelectrochemical Water Splitting

**Figure 6:**
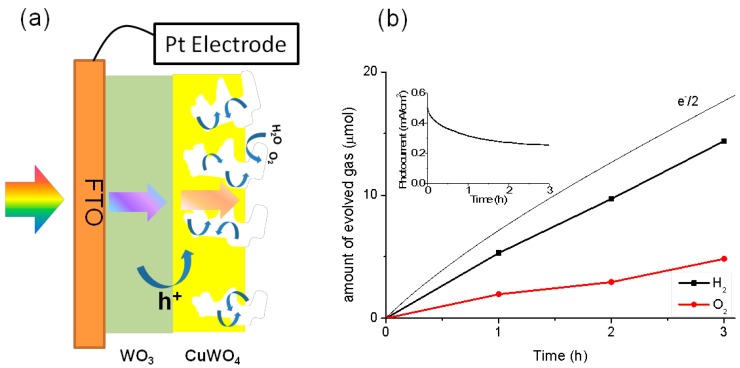
(**a**) Illustration of working mechanism of WO_3_/CuWO_4_ composite photoanode; and (**b**) hydrogen and oxygen evolution by WO_3_/CuWO_4_ photoanode under AM 1.5 illumination in 0.5 M Na_2_SO_4_ at bias of 1.20 V_RHE_. The expected amount of hydrogen gas, e^−^/2 is also provided for evaluation of Faradaic efficiency. Inset graph shows the time course of photocurrent generation in 3 h.

In order to confirm that photocurrent was generated by water splitting, we conducted hydrogen and oxygen evolution under AM 1.5 illumination with 1.20 V_RHE_ in 0.5 M Na_2_SO_4_ electrolyte. Figure 6a illustrates how the charge carriers are transported in the WO_3_/CuWO_4_ composite photoanode. Both WO_3_ and CuWO_4_ are excited by back illumination and generate charge carriers. Holes from WO_3_ are transferred to CuWO_4_ due to the formation of heterojunction, and are injected into the electrolyte from porous CuWO_4_ surface to oxidize water into O_2_. Electrons are directed to the Pt electrode where water molecules are reduced to hydrogen gas. As shown in Figure 6b, the total amount of oxygen and hydrogen evolved in three hours is about 5.0 and 14.0 μmol, respectively. The ratio of H_2_ to O_2_ produced is greater than the stoichiometric ratio [36]:

Faradaic Efficiency (FE) % = actual hydrogen evolution rate/calculated amount from photocurrent generation × 100
(5)

which was about 79% using the hydrogen quantity calculated according to Equation (5). The loss of faradaic efficiency was possibly due to the slow kinetics of water oxidation and back reaction of H_2_ and O_2_. The 3 h time course photocurrent density is presented in the inset of Figure 6b. The photocurrent dropped to around 63% of the initial current density within the first hour, but we believe that the stability of WO_3_/CuWO_4_ photoanode could be improved by loading oxygen evolution reaction (OER) co-catalyst on the electrode surface.

## 3. Materials and Methods

### 3.1. Preparation of W Thin Film from Magnetron Sputtering

**Scheme 1:**
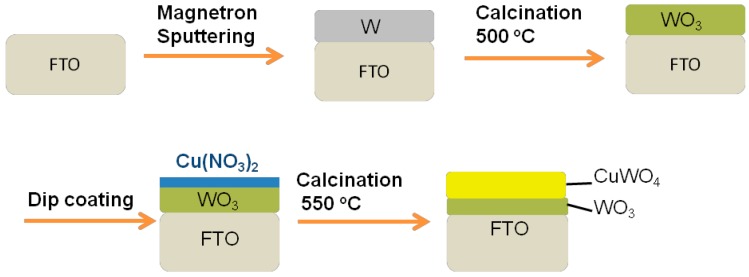
Preparation of FTO/W, FTO/WO_3_, FTO/WO_3_/Cu^2+^ and FTO/WO_3_/CuWO_4_ thin films.

The synthesis route of the WO_3_/CuWO_4_ film is shown in Scheme 1. Tungsten film was deposited onto F-doped tin oxide (FTO) glass (sheet resistance ≤ 15 Ω/square, size: 10 mm × 25 mm and thickness: 2.2 mm) using direct current (DC) magnetron sputtering. The FTO glass was cleaned using acetone, ethanol and DI water prior to the sputtering. A metallic tungsten target (W, 3.00′′ diameter × 0.250′′ thick, 99.95% purity, Kurt J. Lesker, Jefferson Hills, PA, USA) was used as the sputtering target. The distance between the target and the substrate was set at around 10 cm. The sputtering chamber was evacuated to 8.0 × 10^−6^ Torr or lower using a rotary pump and a turbo pump before introducing argon gas. The argon flow rate was kept constant at 20 sccm. A manual gate valve was used to fix the pressure inside the sputtering chamber at 20.0 mTorr during film deposition. The surface of the target was cleaned by sputtering the W target for 10 min before deposition onto FTO glass. The whole sputtering process lasted for 5.0 min at a constant working power of 230 W to obtain the black metallic W thin film.

### 3.2. Fabrication of WO_3_ Thin Film

The as-prepared metallic W thin film was placed in a clean porcelain crucible, and was transferred to a muffle furnace (CWF 12/5, Carbolite, Derbyshire, UK). The thin film was calcined at 500 °C for 2 h with a ramping rate of 2 °C/min for heating step and was cooled down naturally. Monoclinic WO_3_ thin film with a light yellow colour was obtained.

### 3.3. Fabrication of WO_3_/CuWO_4_ Thin Films

0.2 M of copper nitrate trihydrate (Cu(NO_3_)_2_∙3H_2_O, Sigma-Aldrich, St. Louis, MO, USA) ethanol solution with 0.04 g/mL of Pluronic^®^ F-127 (Sigma-Aldrich, St. Louis, MO, USA) was prepared as the dip coating solution. The FTO/WO_3_ was installed on dip coater (KSV NIMA), with insertion and withdrawal speed of 100 mm/min and 30 s submerge duration in Cu(NO_3_)_2_∙3H_2_O ethanol solution. The process was repeated for 4 and 8 runs to load different amount of Cu^2+^ ions. The films were subsequently heated in air at 550 C for 4 h in a muffle furnace, with a ramping rate of 2 °C/min for both heating and cooling steps. After annealing, the colour of the thin film changed to bright yellow, indicating the formation of a layer of CuWO_4_ during heat treatment. CuO that formed from decomposition of excess Cu(NO_3_)_2_ along the edges of WO_3_ thin film and FTO glass during heating was dissolved away by soaking in a 0.5 M HCl solution for 10 min.

### 3.4. Material Characterization

X-ray diffraction (XRD) patterns of all the thin films were recorded using XRD-6000 X-ray diffractometer (Shimadzu, Kyoto, Japan) with Cu Kα radiation (λ = 0.15418 nm). The surface morphology was analyzed by FESEM (JSM-7600F, JEOL, Tokyo, Japan). The morphology of CuWO_4_ nanoparticles were observed using TEM (JEM-2010, JEOL, Tokyo, Japan).UV-Vis/DRS of thin films was recorded using a Varian Cary 5000 UV-Vis spectrophotometer (Varian/Agilent, Santa Clara, CA, USA), with BaSO_4_ as a reference. Absorption efficiency (η_abs_) of thin films was determined by measuring transmission and reflection spectra using integrating sphere (Absorbance = 1 − Transmittance − Reflectance) [37].

### 3.5. Photoelectrochemical (PEC) Measurement

To investigate the photoelectrochemical properties of WO_3_, WO_3_/CuWO_4_, and CuWO_4_ photoanodes, a conventional three-electrode system was used. All the PEC measurements were carried out in a home-made Teflon PEC cell with an illumination window of 5 mm inner diameter for back-illumination. The surface area of electrode exposed under illumination was about 0.2 cm^2^. 0.5 M of sodium sulphate (Na_2_SO_4_, Sigma-Aldrich, St. Louis, MO, USA) in deionized water solution with a pH value of 6 was used as the electrolyte. Platinum (Pt) coil and silver/silver chloride (Ag/AgCl) were used as counter and reference electrodes, respectively. The prepared thin films were used as working electrodes. The light source was simulated sunlight from a 150 W xenon solar simulator (67005, Newport Corp., Irvine, CA, USA) through an Air Mass filter (AM 1.5, Global, 81094, Newport Corp., Irvine, CA, USA) with a constant light intensity to standard AM1.5 sunlight (100 mW/cm^2^) at the photoanode surface. Linear sweep voltammetry (LSV) was carried out by an electrochemistry workstation (CHI 852C, CH Instruments, Shanghai, China) both in dark and under AM1.5 sunlight simulator. Stability of WO_3_/CuWO_4_ sample was carried out at a bias potential of 1.20 V_RHE_ for 600 s with illumination on-off interval of 5 s. Charge separation efficiency was obtained by measuring light absorption of thin films and photocurrent in 0.5 M Na_2_SO_4_ + 0.5 M H_2_O_2_ aqueous solution. Incident photon to electron conversion efficiency (IPCE) was measured with a xenon light source (66983, Newport Corp., Irvine, CA, USA) coupled with a monochromator (74125, Newport Corp., Irvine, CA, USA) at a bias of 1.20 V_RHE_ from back illumination. A Si photodiode (DH-Si, Bentham, Reading, Berkshire, UK) with known IPCE was used to calculate the IPCE of prepared thin films. A source meter (Keithley Instruments Inc., Solon, OH, USA, Model: 2400) was used to record the photocurrent of Si diode. CHI 852C electrochemistry workstation was used to record the photocurrent of each photoanode. IPCE calculation is given in the following formula:

IPCE (λ) = 100 × 1240 × (J(λ) − J_dark_)/λ/I(λ) [38] (6)

where λ is the wavelength of light in unit of nm; J(λ) is the photocurrent density in mA/cm^2^ under illumination at λ; J_dark_ is the photocurrent density measured at dark; and I(λ) is the incident light intensity in mW/cm^2^ at λ [38].

Electrochemical impedance spectroscopy (EIS) was performed using an Autolab PGSTAT 302N system (Metrohm Autolab, Utrecht, The Netherlands) equipped with the FAR2 Faraday impedance module (Metrohm Autolab, Utrecht, The Netherlands). The flat band potential of CuWO_4_ was determined using the Mott-Schottky equation on a CuWO_4_ sample at frequencies of 5 k and 10 k Hz. The Nyquist plot was measured at 1.20 V_RHE_ with a frequency ranging from 0.01 Hz to 100 kHz at 10 mV amplitude potential under AM1.5 illumination.

### 3.6. Photoelectrochemical Water Splitting

The photoelectrochemical water splitting was carried out in an air-tight reactor using back illumination. The light source (AM 1.5 sunlight, 100 mW/cm^2^) and applied bias 1.20 V_RHE_ were kept the same during the measurement. The amount of hydrogen and oxygen was analyzed by a gas chromatographer (GC-7890A, Agilent, Agilent, Santa Clara, CA, USA) equipped with thermal conductivity detector (TCD) detector.

## 4. Conclusions

In conclusion, we have converted monoclinic WO_3_ thin film into WO_3_/CuWO_4_ composite and CuWO_4_ films through a facile dip-coating step followed by heat treatment. The PEC and EIS measurements showed that the presence of a thin layer of WO_3_ beneath CuWO_4_ can enhance the photocurrent density and reduce the charge transfer resistance compared with pure CuWO_4_ film. By separately studying the photo absorption and charge transfer efficiencies, it was demonstrated that the WO_3_/CuWO_4_ composite film exhibited enhancements in each process compared with single-phase WO_3_ and CuWO_4_. Hydrogen and oxygen evolution was conducted to confirm that the photocurrent was generated from water splitting. Our result has provided a low-cost and controllable method to prepare WO_3_-metal tungstate heterojunction thin films, and helped to provide a reference for designing CuWO_4_-based photoanodes with greater efficiency.

## Figures and Tables

**Table 1:** Summary of the thickness of the WO_3_ and CuWO_4_ layers of the three samples.

Sample	Thickness of WO_3_ (nm)	Thickness of CuWO_4_ (nm)
Pristine WO_3_	415	0
WO_3_/CuWO_4_	150	600
CuWO_4_	0	1000

## Supplementary Materials

The following are available online at www.mdpi.com/1996-1944/9/5/348/s1. Figure S1: XRD patterns of different WO_3_ phases obtained from magnetron sputtering on FTO (red) and normal glass slide (blue) substrates, which showed the FTO layer helped to induce the crystal growth of monoclinic WO_3_. Figure S2: TEM images of particles scraped from WO_3_/CuWO_4_, indicating network morphology of the CuWO_4_ layer. Figure S3: Photocurrent comparison of thin film obtained from different runs of dip coating. Figure S4: Mott-Schottky plots of WO_3_/CuWO_4_ thin film at 10 k and 5 k Hz under dark conditions. Figure S5: Absorption efficiency of the WO_3_, CuWO_4_ and WO_3_/CuWO_4_ thin film by measuring the transmission and reflection spectra using an integrating sphere (Absorbance (η_abs_) = 1 − Transmittance − Reflectance). Figure S6: Linear sweep voltammetry of all samples with (solid lines) and without the illumination of AM 1.5 (dashed lines), measured in 0.5 M Na_2_SO_4_ + 0.5 M H_2_O_2_ aqueous solution.
