# Supplementary material for: Improved Charge Separation in WO3/CuWO4 Composite Photoanodes for Photoelectrochemical Water Oxidation
Source: Materials (Basel). 2016 May 7;9(5):348. doi: 10.3390/ma9050348 (PMC5503067; doi:10.3390/ma9050348)
Supplement: Supplementary file 1 [file materials-09-00348-s001.pdf]

Supplementary Materials: Improved Charge Separation in $WO_3/CuWO_4$ Composite Photoanodes for Photoelectrochemical Water Oxidation

Danping Wang, Prince Saurabh Bassi, Huan Qi, Xin Zhao, Gurudayal, Lydia Helena Wong, Rong Xu, Thirumany Sritharan and Zhong Chen

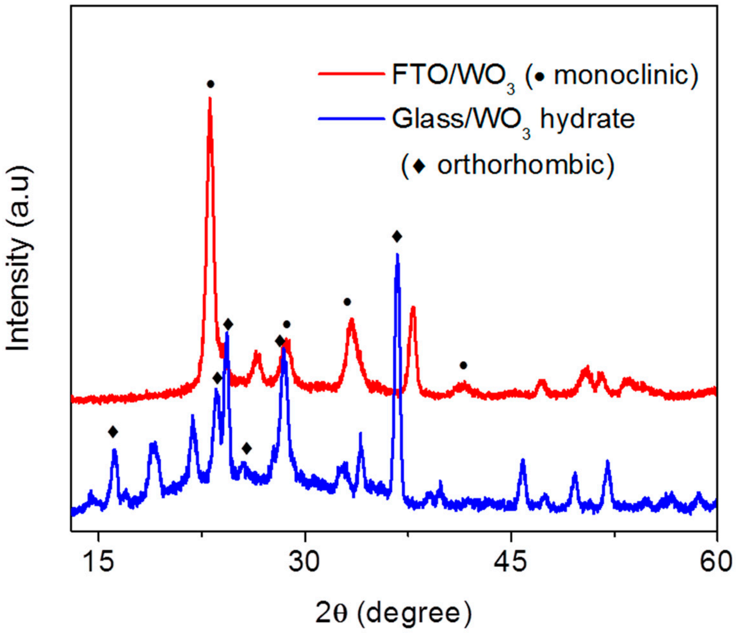

Figure S1. XRD patterns of different WO_3 phases obtained from magnetron sputtering on FTO (**red**) and normal glass slide (**blue**) substrates, which showed the FTO layer helped to induce the crystal growth of monoclinic WO_3.

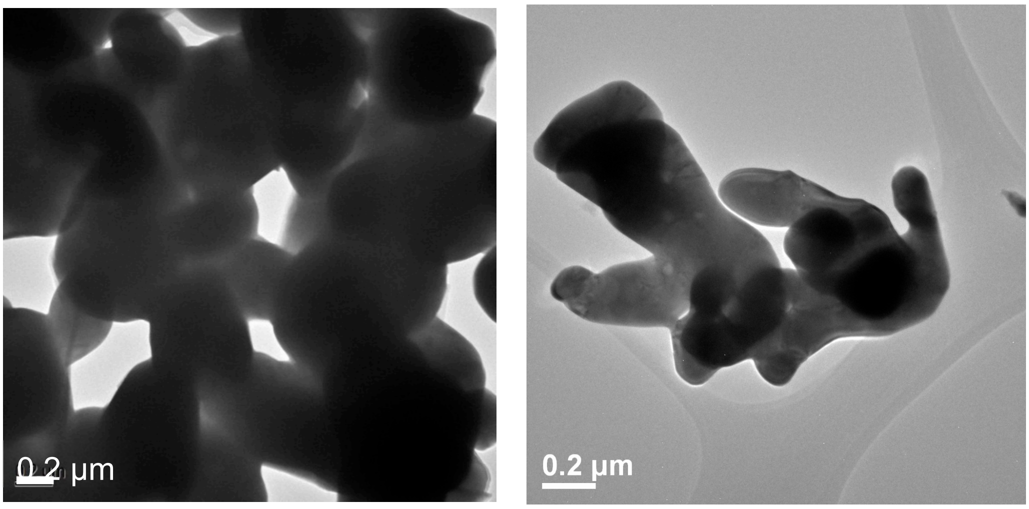

(**a**) (**b**)

Figure S2. TEM images of particles scraped from $WO_3/CuWO_4$, indicating network morphology of the $CuWO_4$ layer. (**a**) Network structure of $CuWO_4$ layer; (**b**) Branched $CuWO_4$ nanoparticles from broken network piece.

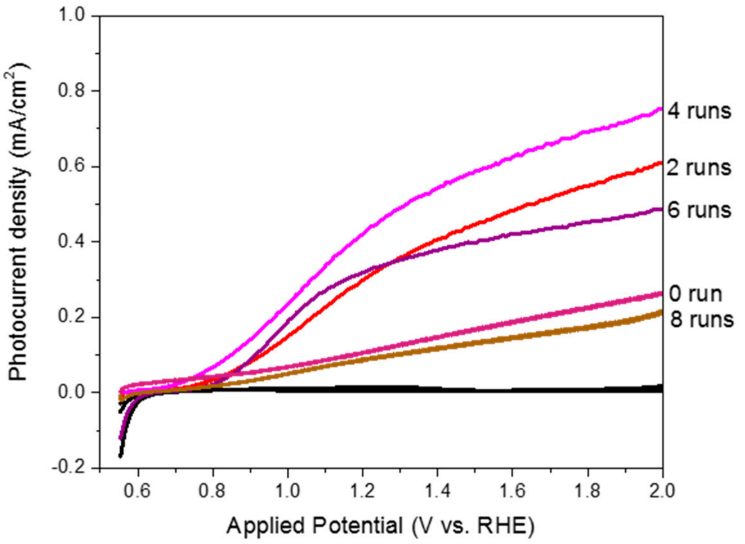

Figure S3. Photocurrent comparison of thin film obtained from different runs of dip coating. (Colored lines: Photocurrent under AM 1.5G illumination, Black lines: dark current).

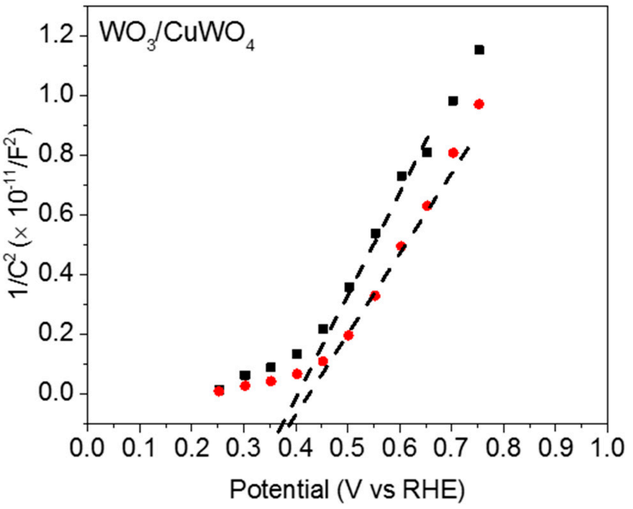

Figure S4. Mott-Schottky plots of $WO_3/CuWO_4$ thin film at 10 k (black) and 5 k Hz (red) under dark condition.

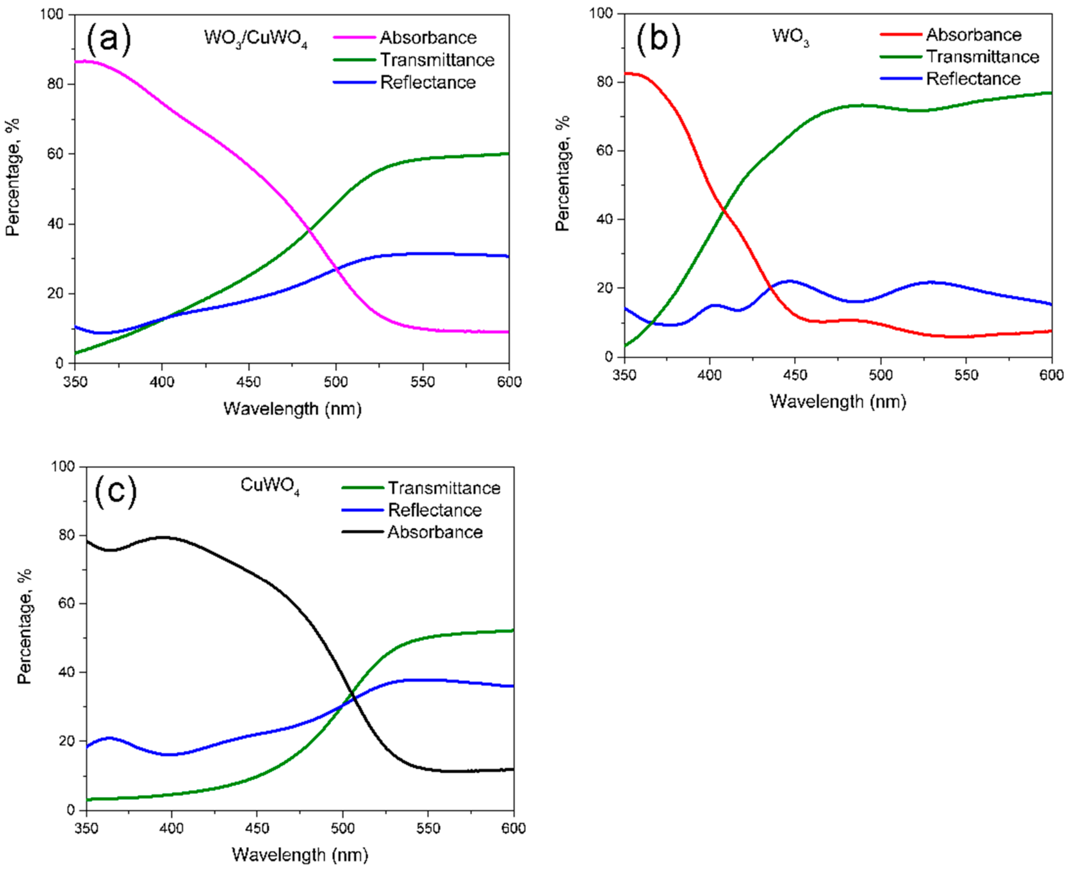

Figure S5. Absorption efficiency of: (**a**) $WO_3/CuWO_4$; (**b**) WO_3; and (**c**) $CuWO_4$ thin films by measuring the transmission and reflection spectra using an integrating sphere (Absorbance (η_{abs}) = 1 − Transmittance − Reflectance).

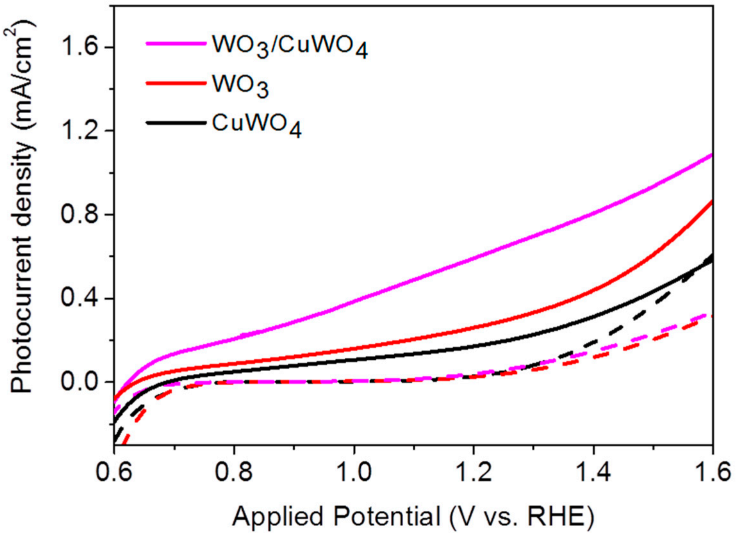

Figure S6. Linear sweep voltammetry of all samples with (solid lines) and without the illumination of AM 1.5 (dashed lines), measured in 0.5 M Na_2SO_4 + 0.5 M H_2O_2 aqueous solution.
